# When Muscle Lines Blur: An Unusual Fusion of Trapezius and Sternocleidomastoid With Surgical Relevance

**DOI:** 10.7759/cureus.96106

**Published:** 2025-11-04

**Authors:** Vinay Sharma, Padamjeet Panchal, C.S. Ramesh Babu

**Affiliations:** 1 Anatomy, Muzaffarnagar Medical College, Muzaffarnagar, IND; 2 Anatomy, All India Institute of Medical Sciences, Patna, IND

**Keywords:** accessory nerve, agenesis, anatomical variation, cleido-occipitalis, embryology, muscular anomaly, posterior triangle, sternocleidomastoid, supernumerary head, trapezius

## Abstract

During the routine dissection of the cadaver of a 57-year-old Indian woman, a rare constellation of muscular variations was observed in the left cervical region. A well-developed sternocleido-occipital muscle with distinct sternal and clavicular heads arises from the external occipital protuberance, the medial third of the superior nuchal line, the ligamentum nuchae, and the spinous processes of C7-T12. Adjacent to it, a separate cleido-occipital slip merged posteriorly with the main muscle, while an accessory trapezial bundle coursed inferolaterally to the clavicle. Notably, the external jugular vein (EJV) passes between the trapezial bundle and the principal muscle, and a musculotendinous arch overlies the clavicular branch of the supraclavicular nerves, potentially causing compression. These variants collectively altered the configuration of the posterior cervical triangle without evidence of trauma or surgical change. Embryologically, the anomaly likely reflects incomplete separation of the cucullaris anlage, and phylogenetically, persistence of a primitive muscular pattern. Awareness of such configurations is vital, as they may affect surgical, radiological, and anesthetic interventions involving the posterior triangle.

## Introduction

The trapezius, the most expansive superficial muscle of the upper back and cervical region, is classically divided into descending, transverse, and ascending parts, each contributing distinct functions to shoulder and neck mechanics [1,2]. It arises from the external occipital protuberance, the medial third of the superior nuchal line, the ligamentum nuchae, and the spinous processes of C7-T12, reflecting its broad attachment to the posterior axial skeleton [3]. Reported causes for the absence of skeletal muscles include inflammation, vascular anomalies, trauma, and congenital syndromes. The spinal accessory nerve supplies both the trapezius and sternocleidomastoid (SCM) muscles. Anatomical variations of the trapezius encompass a spectrum of anomalies, including partial or total agenesis, reported unilaterally or bilaterally and sometimes linked with absent neural or vascular supply [4-6]. Cases of hypoplasia have also been described, as well as accessory slips capable of compressing adjacent structures like the brachial plexus, producing neurological symptoms [7,8]. Other variants include isolated or separated muscle portions and atypical innervation patterns where the trapezius is supplied solely by the cervical plexus rather than the accessory nerve [9,10]. The familial pattern of occurrence, along with links to Poland and Klippel-Feil syndromes, implies a genetic or developmental pathogenesis [11].

Both the trapezius and SCM muscles may extend unusually along the clavicle, occasionally creating an opening through which the external jugular vein (EJV) passes. Such variations can predispose the vein to compression during shoulder movements or muscle hypertrophy, mimicking vascular disorders. An exceptional case was reported in cadaver of a 60-year-old man, where the SCM and trapezius were fused into a single muscular sheet, termed the “sternocleido-mastoido-occipitalis”, obliterating the posterior triangle and obscuring key landmarks. Embryologically, this anomaly arises from the failure of the sixth arch mesoderm segmentation; phylogenetically, it reflects the persistence of the ancestral cucullaris. Clinically, unawareness of such variants may complicate central venous access, lymph node excision, or reconstructive flap surgery [12,13]. Various hypotheses have been suggested in the literature to account for the absence of skeletal muscles, including factors such as inflammation, vascular anomalies, trauma, and congenital developmental disorders. Among these, particular attention has been given to the role of the accessory nerve, which provides motor innervation to both the trapezius and SCM muscles. Injury or developmental disruption of this nerve can lead to defective or incomplete formation of these muscles, potentially resulting in their partial or complete absence [4]. The classical concept of nerve-muscle specificity is explained by three principles: the Law of Migration, where a muscle migrates from its original region with its nerve (e.g., diaphragm with the phrenic nerve); the Law of Fusion, where dual innervation reflects fusion of two distinct muscle masses (e.g., adductor magnus); and the Law of Separation, where a single mass splits but retains common innervation, as seen in the SCM and trapezius - “brother muscles” supplied by the spinal accessory nerve [14].

## Case presentation

During the routine dissection of a formalin-fixed cadaver in the anatomy laboratory, we identified a variant morphology of the trapezius and SCM muscles on the left side of a 57-year-old North Indian woman. There was a reduction in the anatomical space of the posterior triangle. Dissection revealed that within the superficial cervical fascia, a well-developed sternocleido-occipital muscle, formed by distinct clavicular and sternal heads, converges in the lower neck and inserts into the superior nuchal line and mastoid part of the occipital bone. Adjacent to it, a separate cleido-occipital slip arose from the clavicle, lateral to the clavicular head, later merging posteriorly with the main muscle. Its size was 4.3 cm long, 0.8 cm wide, and 0.6 cm thick. Examination revealed no surgical or traumatic alterations in the region. The finding was documented repeatedly through photographs and measurements obtained with a sliding vernier calliper accurate to 1 mm during dissection. Both the greater occipital nerve and the occipital artery were observed, consistent with their typical emergence and course in the suboccipital region.

**Figure 1:**
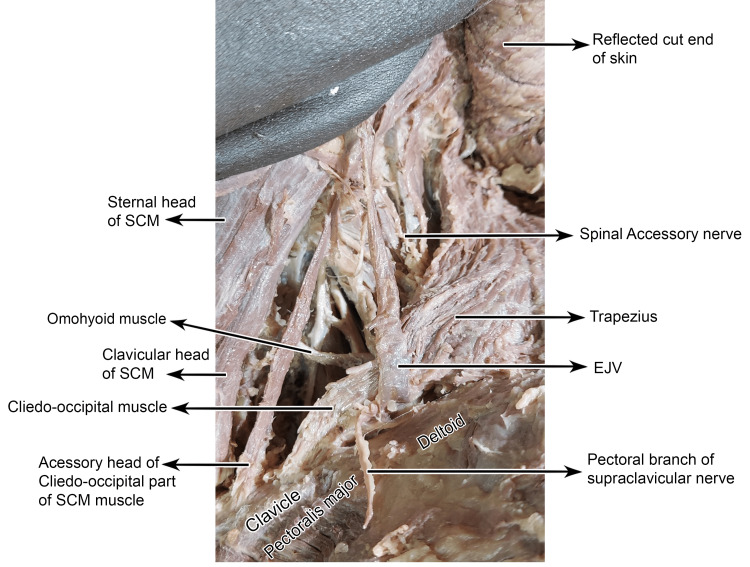
Detailed dissection of the left posterior cervical triangle reveals an accessory muscular slip of the cleido-occipital part of the SCM and a cleido-occipital bundle of the trapezius EJV: External jugular vein; SCM: Sternocleidomastoid

**Figure 2:**
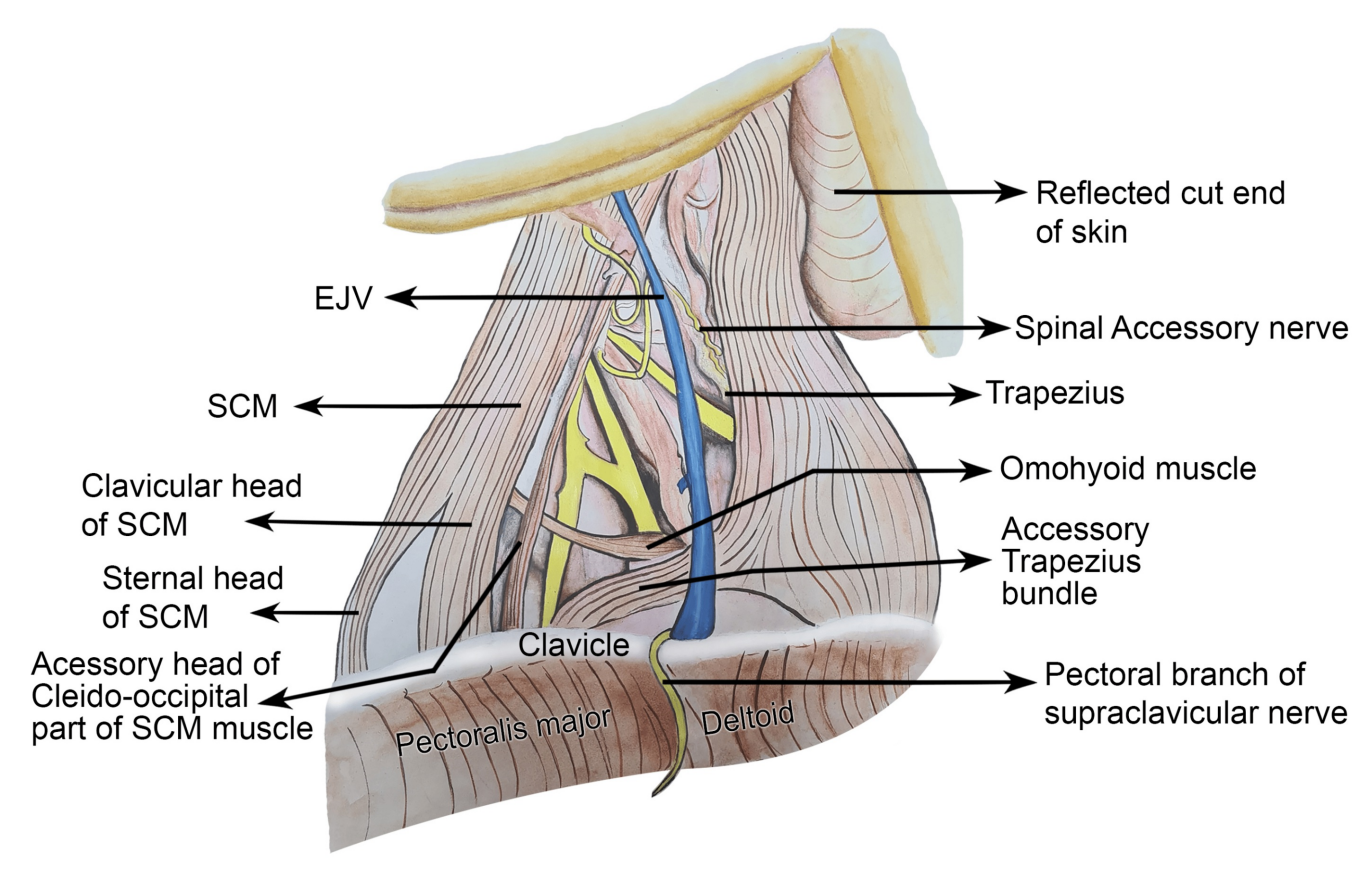
Schematic representation of the left posterior cervical triangle illustrating the accessory cleido-occipital slip of the SCM and the cleido-occipital bundle of the trapezius with their neurovascular relations The illustration depicts the detailed topographical relationship of the accessory cleido-occipital slip of the SCM and the cleido-occipital bundle of the trapezius within the left posterior cervical triangle. The relative course of the spinal accessory nerve and supraclavicular nerves is shown to highlight their proximity to these anomalous muscular slips. Illustration credit: Padamjeet Panchal EJV: External jugular vein; SCM: Sternocleidomastoid

The origin of the trapezius was traced to the occipital bone, the ligamentum nuchae, and the spinous processes of C7-T12 as well as their supraspinous ligaments. The trapezius was inserted into the lateral third of the clavicle, the acromion, and the spine of the scapula, maintaining its typical insertion pattern. In addition to this normal insertion, a distinct muscular bundle was identified as an anatomical variation. The accessory bundle was distinctly set apart from the clavicular fibres of the trapezius by an intervening cleft. It coursed inferolaterally as an isolated slip, terminating in a musculotendinous attachment to the superoposterior aspect of the clavicle medial to the standard trapezius insertion. Importantly, the anomalous bundle and the principal trapezius attachment were separated by the EJV, which passed between them through a cleft. The course of the clavicular branch of the supraclavicular nerves was overlain by a musculotendinous arch, potentially narrowing its passage (Figure 1), and a schematic illustration complementing your dissection photograph. These variants collectively altered the configuration of the posterior cervical space without evidence of trauma or surgical change (Figure 2).

## Discussion

Muscular variations of the SCM and trapezius are of considerable anatomical, embryological, and clinical importance, with reported anomalies ranging from supernumerary heads and accessory slips to partial or complete absence, as well as persistence of fused unseparated muscles. The present case describes a rare coexistence of variant morphologies involving both the SCM and trapezius muscles in the cadaver of a 57-year-old woman. A well-developed sternocleido-occipital muscle, a separate cleido-occipital slip, and an accessory trapezial bundle were identified, collectively leading to a reduction in the posterior cervical triangle. The EJV and supraclavicular nerves were closely related to these anomalous structures, raising important clinical implications.

Comparison with reported literature

Previous authors have documented muscular slips in the posterior triangle, often linking them to potential neurovascular entrapment. Paraskevas et al. reported a clavicular slip approximately 1.6 cm lateral to the SCM origin, and inserted into the medial portion of the upper trapezius, blending with its fibres, directly overlying the supraclavicular nerves [15]. Kwak et al. documented a cleido-occipitalis cervicalis slip extending from the clavicle to the occipital bone, also crossing the supraclavicular nerves [10]. Both cases highlight how anomalous bands can endanger superficial nerve trunks within the posterior triangle. Our case parallels these findings but differs in the coexistence of multiple distinct anomalies, including an isolated trapezial slip that is separated from its main fibres by the EJV, a feature not previously emphasised in prior reports. 

Aragão et al. observed bilateral supernumerary clavicular heads of the SCM muscle in a 23.9-week-old male foetus. Each SCM displayed an additional head arising from the clavicle’s medial third, creating an extra minor supraclavicular fossa on both sides. On the right, the three heads united near the hyoid bone at 22.6 mm from the origin, while on the left, the union occurred at 20.2 mm. Narrowing of the supraclavicular fossae may complicate central venous catheterisation [16]. Fulmali et al. observed a variant left SCM with a third clavicular head in the cadaver of a 70-year-old man. This supernumerary head, arising from the middle third of the clavicle, ascended parallel to the normal clavicular head and fused with the main muscle belly, inserting on the mastoid process. It overlapped key neurovascular structures, including the lesser occipital, supraclavicular, and spinal accessory nerves, while the right side was normal [17]. Kim et al. reported a rare bilateral variation of the SCM in the cadaver of a 67-year-old Korean man, with four heads on each side. On the right, two sternal and two clavicular heads (cleido-occipital and cleido-mastoid) were present, while the left showed one sternal and three clavicular heads with similar components. These supernumerary heads converged into the main belly, inserting on the mastoid process and occipital bone. Such bilateral multiplicity is extremely uncommon and carries significant clinical relevance for cervical surgical and anesthetic procedures [18]. Coskun et al. described a rare unilateral anomaly in the right side of the cadaver of a 25-year-old man, consisting of multiple variant muscles. The superficial layer showed an SCM muscle with distinct clavicular and sternal heads inserting into the superior nuchal line, accompanied by a separate sternomastoid extending from the manubrium to the mastoid. An additional muscular bundle, measuring 5.6 cm, coursed obliquely between them, a feature not previously reported. In the deep layer, the cleidomastoid muscle arose from the sternoclavicular joint, dividing into medial and lateral slips that inserted onto the mastoid process. This constellation of variations represents a unique and previously undescribed anatomical pattern [19]. Singh et al. reported a rare case in the cadaver of a 60-year-old man where the left posterior triangle was obliterated entirely due to fusion of the SCM and trapezius into a broad fasciomuscular sheet encircling the neck. This sheet originated from the manubrium, clavicle, acromion, and scapular spine, and was inserted into the mastoid process, superior nuchal line, external occipital protuberance, ligamentum nuchae, and thoracic spines. Neurovascular structures typically found in the posterior triangle were displaced deep to this fused lamina. An additional oval deficiency (6-8 × 2 cm) below the superior nuchal line exposed the splenius capitis and levator scapulae, while the right side retained normal anatomy. There is a wide morphological spectrum of the SCM-trapezius complex. From supernumerary heads and accessory slips to complete fusion, obliterating the posterior triangle, the literature highlights the considerable variability of these muscles. Such diversity underscores their shared embryonic origin and the potential clinical implications when these variations alter the topography of neurovascular structures [12].

Emsley and Davis described a partial unilateral absence of the trapezius in the cadaver of an 89-year-old man cadaver, most marked in the inferior third of the left muscle. The trapezius terminated at T8-T9 instead of T12, exhibited reduced thickness, and covered an area nearly 50% smaller than the contralateral side. As the accessory nerve and surrounding muscles were normal, the anomaly was considered developmental rather than acquired [8]. Rahman and Yamadori reported a rare bilateral trapezius anomaly in the cadaver of a 68-year-old Japanese woman. The occipital portion was separated to form distinct cleido-occipitalis muscles, accompanied by partial deficiency of upper cervical fibers bilaterally. On the right, the cleido-occipitalis blended with cervical fibers and inserted normally into the clavicle, while on the left, it partially fused with the cervical portion, with some conjoint fibers forming an accessory slip that crossed the posterior triangle to insert as a slender tendon into the posterior clavicle. The anomaly was attributed to secondary degeneration of part of the trapezius anlage and abnormal segregation of another portion during development [20]. 

Nyemb et al. conducted a review of congenital anomalies of the trapezius muscle and noted that, although uncommon (≈approximately 2% incidence), they present in diverse forms. Documented anomalies include unilateral or bilateral agenesis, hypoplasia, partial absence, supernumerary slips, and aberrant innervation, sometimes causing restricted head, neck, or shoulder movement. Some anomalies were associated with syndromes such as Poland and Klippel-Feil, while others appeared as isolated findings in cadavers or imaging studies. Importantly, the review emphasized that even asymptomatic variants have surgical relevance, as they may complicate trapezius flap procedures or be misinterpreted as pathological findings in radiology. Similarly, trapezius anomalies further highlight the developmental instability of this muscle. These variations, though rare, emphasize the plasticity of the trapezius during morphogenesis and its susceptibility to anomalous differentiation. The present case presents a unique pattern combining SCM and trapezial slips, narrowing the posterior cervical triangle with partial separation [21].

Araujo et al. highlighted a spectrum of anomalous interconnections between the SCM and trapezius muscles, resembling the supernumerary bundles. During the dissection, bilateral but asymmetric supernumerary muscle bundles were identified in the posterior cervical region of the cadaver of a middle-aged man. Each fusiform bundle is extended transversely between the upper third of the trapezius and the upper third of the SCM, forming a muscular bridge between the two. These bundles lie superficially over the superior fibres of the trapezius and cross the nuchal region obliquely, thereby altering the typical configuration of the posterior cervical triangle. Embryologically, the SCM and trapezius derive from the common cucullaris muscle of lower vertebrates; incomplete separation of this primordial mass likely explains the anomalous interconnecting bundles observed in this case [22].

A broad morphological spectrum ranging from supernumerary heads and accessory cleido-occipital slips to complete fusion or agenesis of the SCM-trapezius complex. Most anomalies share a common embryological foundation-incomplete or aberrant separation of the cucullaris muscle during development. Clinically, these variants may lead to neurovascular entrapment (particularly of the spinal accessory and supraclavicular nerves), venous obstruction, or misidentification during imaging and surgery. The summary of the reported similar anomalies is tabulated in Table 1.

**Table 1:** Comparative summary of reported variations involving the SCM, trapezius, and cleido-occipital muscles The table emphasizes the morphologic diversity of the SCM–trapezius complex, its embryologic origin, and the potential surgical, radiologic, and anesthetic implications of such variants. EJV: External jugular vein; SCM: Sternocleidomastoid

Study	Type / Laterality	Description of Anomaly	Embryological/Developmental Explanation	Reported or Potential Clinical Significance
Paraskevas et al. [15]	Unilateral accessory clavicular slip	Clavicular slip ~1.6 cm lateral to the SCM origin, inserting into the upper trapezius overlying the supraclavicular nerves.	Variant derived from common SCM–trapezius (cucullaris) primordium	May compress or obscure the supraclavicular nerves during surgery or flap dissection
Kwak et al. [10]	Unilateral cleido-occipitalis cervicalis	The trapezius muscle extends from the clavicle to the occipital bone, crossing the supraclavicular nerves.	Persistence of the cleido-occipital element of cucullaris	Possible entrapment of superficial cervical nerves; altered posterior triangle borders
Aragão et al. [16]	Bilateral supernumerary clavicular heads of SCM (fetal case, 23.9 week)	Each SCM with an extra head from the medial clavicle creates a minor supraclavicular fossa.	Accessory myogenic split during SCM differentiation	May have a narrow supraclavicular fossa, complicating central venous access
Fulmali et al. [17]	Unilateral third clavicular head of SCM (left side)	Additional head from mid-clavicle fusing with the main belly; overlapped by the lesser occipital, supraclavicular, and accessory nerves	Duplication of cleido-sternal myoblasts	Risk of neurovascular compression; challenges in neck dissection
Kim et al. [18]	Bilateral multiple-headed SCM (four heads on each side)	Two sternal and two clavicular heads (right); one sternal and three clavicular heads (left), converging to the mastoid	Excessive segmentation of the SCM anlage	May distort surgical landmarks, increase anesthetic block failure risk
Coskun et al. [19]	Complex multilayered SCM–trapezius variant (right side)	Sternocleido-occipital muscle with distinct heads; an additional 5.6 cm oblique bundle between layers; a separate cleidomastoid slip	Incomplete separation of SCM–trapezius primordia	Alters posterior triangle; risk to the accessory nerve and vascular structures
Singh et al. [12]	Bilateral SCM–trapezius fusion sheet	Complete fusion forms a broad fascia-muscular lamina encircling the neck; the posterior triangle is obliterated	Failure of the separation of common cucullaris	Displacement of the neurovascular bundle: a surgical hazard in the posterior triangle
Emsley & Davis [8]	Partial unilateral absence of trapezius (left side)	Inferior third absent; termination at T8–T9; 50% smaller than contralateral	Developmental trapezial deficiency	Impairs flap harvest, shoulder mechanics
Rahman & Yamadori [20]	Bilateral cleido-occipitalis formation	Separate occipital portions forming distinct cleido-occipital muscles; an accessory slip crossing the posterior triangle	Abnormal segregation and partial degeneration of trapezial anlage	May narrow the posterior triangle; alter the spinal accessory nerve course
Nyemb et al. [21]	Review of congenital trapezius anomalies	Agenesis, hypoplasia, supernumerary slips, aberrant innervation (≈ 2%)	Developmental instability of trapezial myotome	Surgical and radiologic implications; may mimic pathology
Araujo et al. [22]	Bilateral asymmetric interconnecting bundles	Fusiform bundles between the upper SCM and trapezius, crossing the nuchal region	Incomplete separation of cucullaris	Alters posterior triangle; modifies spinal accessory nerve trajectory
Present Case (2025)	Unilateral accessory cleido-occipital slip of SCM with trapezial bundle	Fusion of the accessory slip of the cleido-occipital bundle with the main part of the SCM, along with the existence of the accessory muscle bundle of trapezius, is distinctly set apart from the clavicular fibres of the trapezius by an intervening cleft, separated by the EJV; narrowing of the posterior triangle.	Secondary segregation of cucullaris-derived fibres; abnormal mesodermal splitting of the cucullaris premuscle mass	Accesory head may alter the operative field, potential risk for accessory nerve or EJV injury

Embryological basis

Embryological studies demonstrate that SCM and trapezius share a common origin from the cucullaris premuscle mass, which later separates during fetal development [23,24]. McKenzie highlighted the composite nature of the sternomastoid-trapezius complex through comparative and embryological studies in mammals and human embryos. He demonstrated their origin from a common premuscle mass associated with the accessory nerve and anterior cardinal vein. The presence of a deep sternomastoid portion, seen in humans, pigs, and dogs, reflects myotomic contribution from the omocervicalis, while its absence in rabbits indicates a purely branchial origin. This dual embryonic contribution explains the variability in motor innervation, which derives from both the spinal accessory and cervical spinal nerves [23]. Cho et al. showed that in six-eight week embryos (11-27 mm), the SCM and trapezius arise from a single mesenchymal condensation, which gradually splits cranio-caudally. By 27 mm, separation was nearly complete, assisted by the growth of adjacent muscles and skeletal elements, with lymphatic development accompanying this process. Incomplete or aberrant splitting may result in persistent interconnecting fascicles or accessory slips, as seen here. Asymmetry and multiplicity of such slips may also reflect secondary remodelling influenced by adjacent skeletal growth, vascular development, or lymphatic expansion [24].

Clinical implications

The clinical importance of such variants lies in their potential to modify the anatomy of the posterior cervical triangle, a region traversed by the spinal accessory nerve, supraclavicular nerves, and major superficial vessels. Anomalous slips may cause compression neuropathies, venous entrapment, or misinterpretation as pathological masses on imaging [25]. They may also complicate surgical approaches for central venous catheterization, regional anesthesia, or reconstructive procedures utilizing trapezius flaps [26]. The musculotendinous arch observed over the clavicular branch of the supraclavicular nerves in the present case exemplifies this risk.

Limitations

This case provides detailed morphometric and photographic documentation of multiple coexisting anomalies, supported by careful dissection and measurement. However, as a cadaveric study, it lacks functional or clinical correlation. Future imaging-based studies and larger cadaveric series are needed to determine the prevalence and clinical significance of such anomalies. 

## Conclusions

The coexistence of a sternocleido-occipital muscle, an accessory cleido-occipital slip, and a trapezial bundle separated by the EJV represents a rare configuration. Incomplete separation or aberrant differentiation of this primordial mass during development may account for the persistence of accessory or bridging fibres between the two muscles. Clinically, recognition of such variants is of paramount importance for radiologists, who might misinterpret these structures on imaging as pathological masses or fibrotic bands. It is also crucial for surgeons performing procedures such as neck dissections, venous catheterization, or reconstructive flap harvests, where unexpected muscular slips can obscure anatomical landmarks or entrap nearby nerves. Hence, documenting this rare coexistence expands the anatomical spectrum of posterior cervical muscle variations and reinforces the need for meticulous dissection, careful radiological interpretation, and intraoperative vigilance in the cervical region. Awareness of such anomalies is essential for anatomists, radiologists, and surgeons, as they may alter the topography of the posterior cervical triangle, affect neurovascular structures, and complicate clinical interventions in the cervical region.
